# Use of ISSR markers to assess the genetic diversity of an endemic plant of Morocco (*Euphorbia resinifera O. Berg*)

**DOI:** 10.1186/s43141-023-00543-4

**Published:** 2023-09-06

**Authors:** Hassane Abd-dada, Said Bouda, Youssef Khachtib, Youssef Ait Bella, Abdelmajid Haddioui

**Affiliations:** Laboratory of Agro-Industrial and Medical Biotechnologies, Faculty of Sciences and Techniques, Sultan Moulay Slimane University, Beni Mellal, Morocco

**Keywords:** *Euphorbia resinifera*, Genetic diversity, ISSR, Polymorphism, Atlas Mountain

## Abstract

**Background:**

*Euphorbia resinifera* is a melliferous, medicinal, and endemic plant to Morocco. Nevertheless, its ecological and genetic diversity still unknown. The objective of this study is to analyze the diversity and genetic structure of Moroccan wild populations of *E. resinifera* using ISSR markers. Twelve natural populations collected from its geographical range in Morocco were analyzed using 14 ISSR primers.

**Results:**

A total of 125 bands were obtained, with polymorphism of 74.81%. The polymorphic information content (PIC), resolving power (Rp), Shannon’s information index (I), and total genetic diversity (Ht) were 0.33, 2.8, 0.35, and 0.21, respectively. The analysis of molecular variance showed that 75.56% of the total variability is present within populations and that 24.44% exists among populations. Also, the analysis showed a very low genetic differentiation between groups of mountain range type (FCT = 0.066), mountain versant type groups (FCT =  −0.024), and altitude groups (FCT =  −0.022). Moreover, the geographical distances between populations are correlated with their corresponding genetic distances according to the Mantel test (*r* = 0.507; *P* < 0.0001).

**Conclusion:**

These results suggest that the population structuring follows a model of isolation by geographical distance. Indeed, the genetic structuring of populations into two groups obtained from PCoA and structure analysis revealed a dependence on the geographical origin of the populations. By contrast, the genetic distances are not correlated with the altitude.

## Background

With nearly 2000 species, the genus *Euphorbia* is the largest of the *Euphorbiaceae* family and the second largest genus of flowering plants in the world [1, 2]. Despite its great vegetative diversity [3], the genus is united morphologically by the possession of a cyathium, a highly reduced inflorescence that resembles a single flower [4]. In Morocco, few species exist, with a notable proportion of them being endemic [5]. *Euphorbia resinifera O. berg* is one among them, growing naturally in the Atlas Mountains, particularly in Beni Mellal and Azilal provinces [5]. The plant covers the mountain in a very discontinuous way from the High Atlas Mountain (Demnat) to the Middle Atlas Mountain (El Ksiba) [6]. It presents a cushioned physiognomy constituted by a bush of juxtaposed stems. On average, the growth in height does not exceed one meter; however, their lateral growth is more important ranging from 0.5 to 2 m, which contributes to the decrease of the soil erosion. The mating system of species is primarily allogamous. Indeed, the Cyathes are arranged by 3 in axillary cyme stalks, towards the end of stems and branches; Cyathes are lateral hermaphroditic, stalked with thick peduncle; the median subsessile and male usually develop first and fall before the maturity of the capsules of the lateral cyathiums. *E. resinifera* employs both sexual and vegetative means of reproduction to ensure the survival and expansion of its population [7]. The yellow flowers of this wild species attract and feed the bees. The beekeepers came from all regions of Morocco for take place near of the *E. resinifera* populations during the flowering season. Thus, with an annual honey production of about 300 tons, it is an important plant of the solidary agriculture in the region. It is well known as a melliferous plant for its high therapeutic and nutritional quality honey [8, 9], which has been recognized as a local product of the region and has been labeled Protected Geographical Indication (PGI) [10].

Additionally, *E. resinifera* was recognized as having a vast therapeutic properties and benefits. In folk medicine, it is used to treat some types of complicated dermatoses, cancer treatment and control glycaemia in type II diabetics [11–13]. In 1975, the resiniferatoxin major element in the latex of *E. resinifera* was identified [14]. This molecule has many potential medical applications, analog to capsaicin but 1000 more potent than it [15]. Moreover, many compounds of the latex of this species are endowed with biological activities as an anticancer [16, 17], antioxidant, antibacterial, antifungal [18, 19], an anti-pain and anti-tuberculosis [20, 21].

Nevertheless, the genetic resources of this species underwent important genetic erosion caused by various factors including the overgrazing, the deforestation provoked by a demographic pressure, voluntary or natural fires, and some fungal diseases attacking the plant. Consequently, emergency measures should be taken to safeguard this wild species. Thus, the safeguard and valorization of these local plant genetic resources constitute not only an imperative but also a major component of the stability of natural ecosystems and their valorization in agro-economic, therapeutic, and environmental perspectives. For that, the description of Moroccan *E. resinifera* populations may help to identify different genotypes and rationalize conservative treatments. Therefore, it has become imperative to establish a research program aiming at the evaluation of the genetic diversity of this species. Our first study on morphological characters showed high phenotypic diversity in twelve Moroccan populations [3]. However, no information is available on its genetic diversity. For this, it becomes necessary to find more discriminating markers, which could provide information about the variability within and among populations of this species and investigate new resources of variation which might be used for specific conservation programs.

Several types of molecular markers are employed for assessment of genetic diversity and relationships in plant species, including RFLPs (restriction fragment length polymorphisms) [22], RAPDs (randomly amplified polymorphic DNAs) [23–25], AFLP (amplified fragment length polymorphisms) [26, 27], SNPs (single-nucleotide polymorphic) [28], SSRs (simple sequence repeats) [29–31], and ISSRs (inter simple sequence repeats) [32–35]. However, RAPDs have low reproducibility, RFLPs are time-consuming and labor-intensive, SSRs require the knowledge of the flanking regions for the development of species-specific primers, and AFLPs and SNPs have high cost [36], while the ISSR markers are hypervariable, highly reproducible, fast, inexpensive, and do not require any prior sequence information of amplified locus [36, 37]. ISSR is a kind of DNA sequences confined by two inverted SSR composed of the same motives, which are amplified by a unique PCR primer. ISSR-PCR detects the levels of variation in microsatellite regions and gives multi-locus schemes, which are very iterative, plentiful, and polymorphic in plant genomes [38, 39].

The present work provides the first data on the genetic diversity of Moroccan *E. resinifera* based on molecular markers. Our objectives were to give a preliminary estimation of the genetic diversity and structure of this species. Then, twelve natural populations of *E. resinifera* originating from diverse altitudes and geographical area were analyzed using fourteen ISSR primers.

## Materials and methods

### Plant material and DNA extraction

**Fig. 1:**
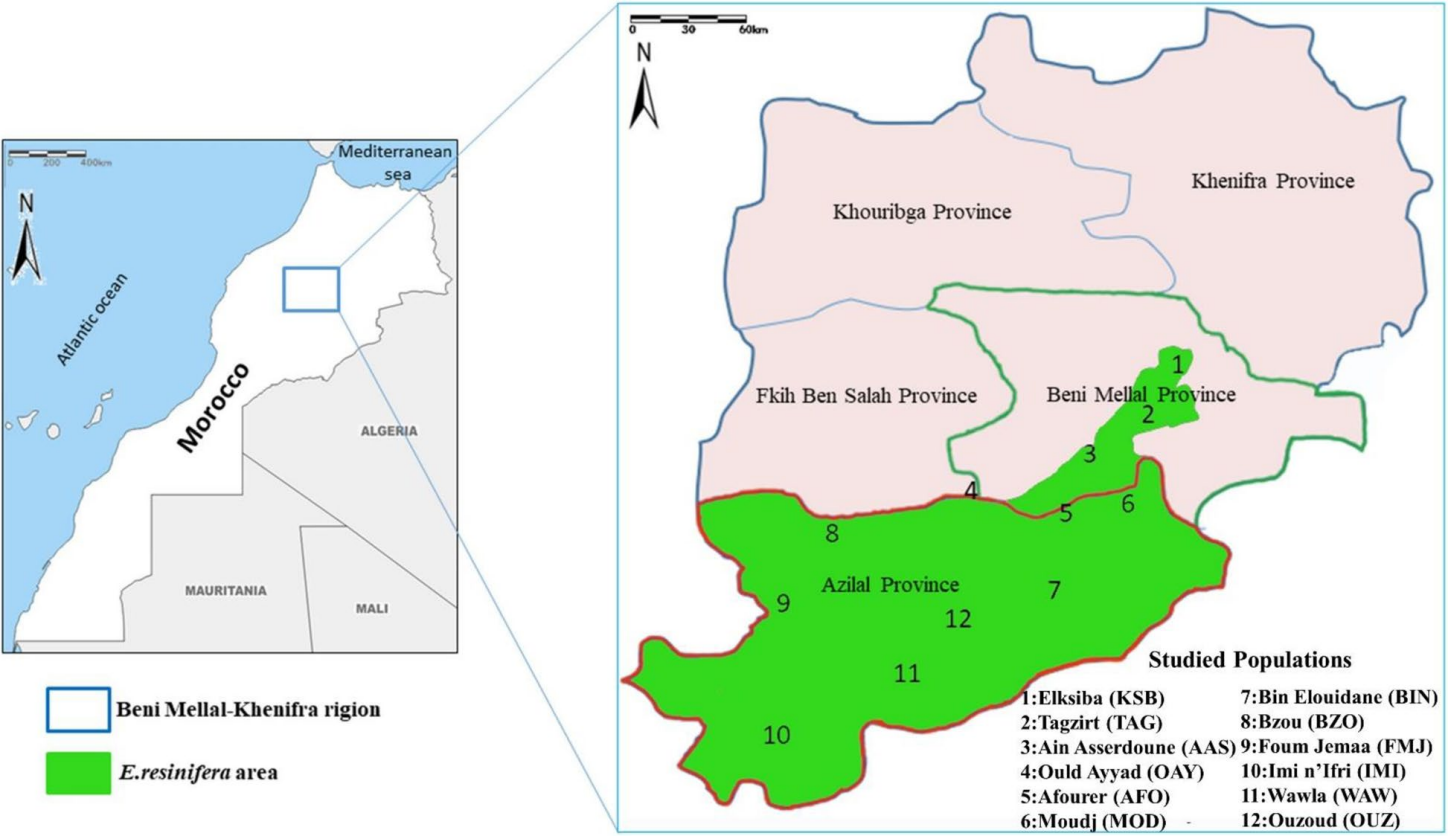
Map of Morocco showing locations of the *E. resinifera* populations studied

The plant material used in this study included twelve natural populations representing the distribution area of *E. resinifera* in Morocco (Fig. 1). Six populations are originating from High Atlas Mountain and six belonging to the Middle Atlas Mountain. The geographic characteristics and meteorological conditions such as altitude slice, central latitude and longitude as well as the mean precipitation and temperature average of these populations are provided in Table 1. From each population, five bush of similar age were randomly sampled and the collected young stems were stored at −20 °C until DNA extractions.

**Table 1:** Geographic and ecological characteristics of *E. resinifera* populations used in the study

Populations	Abbreviations	Geographic origin	Mountain range	Mountain versant type	Latitude N	Longitude W	Altitude (m)	Rainfall average (mm/year)	Temperature average (°C/year)
Elksiba	KSB	1 km N of Elsiba	Middle Atlas	South versant	32° 34′	6° 2′	1045 (low)	718	16.4
Tagzirt	TAG	5 km Northeast of Taghzirt	Middle Atlas	Southwest versant	32° 25′	6° 11′	751 (very low)	550	18.5
Ain Asserdoune	AAS	2 km South of BeniMellal	Middle Atlas	North versant	32° 19′	6° 19′	771 (very low)	493	18.3
Oulad Ayyad	OAY	4 km South of Oulad Ayyad	Middle Atlas	North versant	32° 11′	6° 48′	564 (very low)	506	18.9
Afourer	AFO	6 km South of Afourer	Middle Atlas	North versant	32° 11′	6° 31′	793 (very low)	443	18.8
Modj	MOD	15 km East of Beni Mellal	Middle Atlas	South versant	32° 17′	6° 18′	1223 (low)	550	18.1
Bin El Ouidane	BIN	6 km South west of Bin El Ouidane	High Atlas	North versant	32° 4′	6° 27′	936 (very low)	490	17.6
Bzou	BZO	3 km South of Bzou	High Atlas	South versant	32° 5′	7° 3′	494 (very low)	350	19.3
Foum Jemaa	FMJ	7 km West of Foum Jemaa	High Atlas	Southwest versant	31° 59′	7° 1′	773 (very low)	444	17.4
Imi n’Ifri	IMI	10 km East of Demnat	High Atlas	Southwest versant	31° 43′	6° 58′	1127 (low)	478	16.4
Wawla	WAW	30 km East of Azilal	High Atlas	South versant	31° 54′	6° 43′	1237 (low)	521	16.2
Ouzoud	OUZ	3 km North of Ouzoud	High Atlas	Southwest versant	32° 01′	6° 41′	941 (very low)	456	17.7

The DNA was extracted following the method described by Doyle and Doyle [40] slightly modified [41]. The quality of extracted DNA was examined by electrophoresis on 1% agarose gel, and DNA quantity was determined spectrophotometrically. Later, the samples were brought to a working concentration of 10 ng/μl.

### ISSR analyses

A total of 14 ISSR primers previously displayed reliable and polymorphic band profiles [42, 43] were used in this work (Table 2). The amplification reactions were performed in a volume of 12.5 μl, which contained 6.25 μL 2 × Green Taq Mix (Vazyme, Nanjing, China), 0.5 μL of each primer (0.4 μM), 5 μL of template genomic DNA (4 ng/μL), and 0.75 μL of distilled deionized water. PCRs were conducted in a DNA thermocycler (Multigene gradient, Labnet, NJ. USA). The PCR program consisted of an initial denaturation at 94 °C for 5 min, followed by 35 cycles according to the following procedure: denaturation at 94 °C for 30 s, annealing at determined temperature for 30 s, extension at 72 °C for 1 min, the last cycle was followed by a final extension for 7 min at 72 °C. Gradient PCR was used to determine the annealing temperature of each primer (Table 2). PCR products were separated by electrophoresis on 1.7% agarose gel submerged in 0.5 × TBE buffer and stained with 1 μg/μl of ethidium bromide. The DNAs were visualized under UV light using the Gel Doc system (EnduroTM GDS, Labnet). The fragment size was estimated by using a DNA marker (100 bp plus II DNA ladder, TransGen Biotech Co., Ltd).

**Table 2:** Properties of ISSR markers used in the study and statistical parameters: polymorphism information content and resolving powers

ISSR primer	Sequence (5′-3′)	AT °C	Number of amplified bands		PPB (%)	PIC	Rp
			Total	Polymorphic			
UBC 810	(GA)8 T	44.1	8	4	50.00	0.40	2.23
UBC 811	GA (AG)7C	48.5	12	12	100.00	0.32	4.87
UBC 814	(CT)8A	44.4	10	10	100.00	0.32	3.8
UBC 815	(CT)8A	44.4	8	8	100.00	0.23	2.07
UBC 827	(AC)8G	46.4	11	5	45.45	0.44	2.7
UBC 834	(AG)8YT	44.6	8	4	50.00	0.48	3.13
UBC 836	(AG)8YA	49.1	10	5	50.00	0.35	2.33
UBC 840	(GA)8YT	48.5	13	13	100.00	0.25	3.93
UBC 841	(GA)8YC	47.9	12	4	33.33	0.43	2.33
UBC 843	(CT)8RC	50.2	11	9	81.82	0.29	3.27
UBC 844	(CT)8RC	50.2	12	11	91.67	0.28	3.37
UBC 845	(CT)8RG	44.6	6	4	66.67	0.44	2.37
UBC 853	(TC)8RT	50.2	7	6	85.71	0.14	0.97
UBC 855	(AC)8YT	49.2	7	6	85.71	0.25	1.77
Average			9.64	7.21	74.81	0.33	2.8

*AT°C* Annealing temperature in C°, *Y* (C,T), *R* (A,G), *PPB* Percentage of polymorphic bands, *PIC* Polymorphic information content, *Rp* Resolving power

### Data analyses

The band profiles of each gel were scored visually and recorded as presence (1) or absence (0) of bands leading to the construction of data binary matrix (1,0). For each primer, the percentage of polymorphic band (PPB), the polymorphic information content (PIC), and resolving power (Rp) were determined. Also, the POPGENE software was used to measure the following parameters: numbers of alleles (Na), effective number of alleles (Ne), genetic diversity within populations (Hs), total gene diversity (Ht), coefficient of gene differentiation (Gst), and Shannon’s information index (I). Partition of the observed genetic variation and calculation of the corresponding *F* values were carried out using different hierarchical analysis of molecular variance (AMOVA). Firstly, global analysis of AMOVA was done to apportion the total genetic variation into two hierarchical levels: among populations (FST) and within populations. Secondly, hierarchical AMOVA analysis was used to partition the variation among:

- Two mountain range groups of populations: Middle Atlas (populations: KSB, TAG, AAS, OAY, AFO, and MOD) and High Atlas (populations: BIN, BZO, FMJ, IMI, WAW, and OUZ)
- Three mountain versant groups of populations: North versant (populations: BIN, AAS, OAY, and AFO), Southwest versant (populations: OUZ, TAG, FMJ, and IMI), and South versant (populations: BZO, MOD, WAW, and KSB)
- Two altitude groups of populations: very low (populations: BZO, OAY, TAG, AAS, FMJ, BIN, OUZ, and AFO) and low (populations: KSB, MOD, IMI, and WAW) (Table 1)

Population’s specific FST indices were also calculated to investigate which population is more divergent from the remaining. The average gene diversity over loci was calculated to estimate intra-population variability. These evaluations were performed using the package ARLEQUIN version 3.01 [44]. The pairwise genetic differentiations (FST) between the twelve populations were also generated by AMOVA and the gene flow (N_e_m) was approximated estimated through Wright’s island model: N_e_m = 0.25 (1/FST - 1) [45]. A Mantel test was used to test whether matrix of genetic distances (FST) between populations was significantly correlated with their corresponding matrices of geographic distances and difference of altitude (1000 permutations; routine MXCOMP of the NTSYS-pc; package). A Bayesian structure analysis was executed using the STRUCTURE v.2.3.4 software to deduce the population genetic structure and define the number of groups within the studied populations [46]. To identify the number of K clusters explaining the observed genetic structure, we used the STRUCTURE Harvester website [47], which implements the Evanno method [48]. Furthermore, to elucidate the populations’ relationships, principal coordinate analysis (PCoA) was carried out using the DARwin software version 6.0.02 [49].

## Results

### ISSR polymorphism

The 14 ISSR primers amplified a total of 125 bands in the set of twelve *E. resinifera* populations, of which 101 band were polymorphic. The number of bands ranged from 4 (UBC810, UBC834, UBC84, and UBC845) to 13 (UBC840), with an average of 7.21 (Table 2). The percentage of polymorphic bands (PPB) oscillated from 33.33% (UBC84) to 100% (UBC811, UBC814, and UBC840), with an average of 74.81%. The PIC value varied from 0.14 (UBC853) to 0.48 (UBC834) with mean of 0.33. Regarding, the resolving power (Rp), which present an interesting tool to determine the efficiency of primer to differentiate between populations, varied from 2.07 (UBC815) to 4.87 (UBC811), with an average of 4.91.

### Genetic diversity

Estimates of genetic diversity of studied populations are summarized in Table 3. The results showed that the number of observed alleles (Na) was fixed in the value of 2 for all primers. The highest effective number of alleles (Ne) varied from 1.09 (UBC853) to 1.56 (UC834) with a general mean of 1.34 allele per primer, while Shannon’s Information index (I) showed a lowest value (0.17) for UBC853 and the highest value (0.54) for UBC834, with an average of 0.35. In addition, the total genetic diversity (Ht) oscillated from 0.08 for UBC853 to 0.33 for UBC834 with an average of 0.21. The genetic diversity within species (Hs) ranged from 0.06 for UBC853 to 0.31 for UBC834 (mean 0.15). Moreover, Nei’s coefficient of genetic differentiation between populations (G_ST_) varied from 0.12 (UBC834) to 0.44 (UBC836) with an average of 0.32, indicating that 32% of total genetic variability was distributed among populations and the remaining (68%) accounted for within populations. These results were congruent with that revealed by FST value, accounting for 0. 244 (Table 4). The great level of genetic differentiation among population of this wild plant is in accordance with the low value of gene flow estimated (N_e_m = 0.77) which provides information on amount of number of exchanged individuals between populations studied.

**Table 3:** Genetic diversity revelated by the 14 ISSR primers

ISSR primer	Sample size	Na	Ne	I	Ht	Hs	Gst
UBC 810	60	2	1.39	0.43	0.27	0.20	0.19
UBC 811	60	2	1.33	0.34	0.21	0.10	0.38
UBC 814	60	2	1.23	0.33	0.20	0.11	0.31
UBC 815	60	2	1.20	0.23	0.13	0.07	0.22
UBC 827	60	2	1.48	0.47	0.30	0.21	0.27
UBC 834	60	2	1.56	0.54	0.33	0.31	0.12
UBC 836	60	2	1.38	0.41	0.25	0.13	0.44
UBC 840	60	2	1.23	0.27	0.16	0.09	0.27
UBC 841	60	2	1.48	0.44	0.28	0.23	0.14
UBC 843	60	2	1.24	0.32	0.18	0.13	0.20
UBC 844	60	2	1.44	0.27	0.16	0.11	0.18
UBC 845	60	2	1.50	0.44	0.28	0.22	0.17
UBC 853	60	2	1.09	0.17	0.08	0.06	0.17
UBC 855	60	2	1.18	0.28	0.15	0.11	0.19
Average		2	1.34	0.35	0.21	0.15	0.32

*Na* Number of observed alleles, *Ne* Effective number of alleles, *I* Shannon’s Information index, *Ht* Total genetic diversity, *Hs* Genetic diversity within group, *Gst* Genetic differentiation among group

**Table 4:** AMOVA analysis of the ISSR variation of *E. resinifera* populations

Source of variation	d.f	Sum of squares	Variance components	Percentage of variation	*F*-statistics
**Global**					
Among populations	11	308.267	3.463 Va	24.44	FST = 0.244***
Within populations	48	514.000	10.708 Vb	75.56	
**Hierarchical**					
**a)** Among mountain range type groups	1	54.600	0.974 Va	6.67	FCT = 0.066*
Among populations within groups	10	253.667	2.931 Vb	20.06	FSC = 0.214***
Within populations	48	514.000	10.708 Vc	73.27	FST = 0.267***
**b)** Among mountain versant type groups	2	44.767	−0.344 Va	−2.45	FCT = −0.024 NS
Among population within groups	9	263.500	3.713 Vb	26.38	FSC = 0.257***
Within populations	48	514.000	10.708 Vc	76.07	FST = 0.239***
**c)** Among altitude groups	1	20.417	−0.313 Va	−2.24	FCT = −0.022 NS
Among population within groups	10	287.850	3.615 Vb	25.81	FSC = 0.252***
Within populations	48	514.000	10.708 Vc	76.43	FST = 0.235***
Total	59	822.267	14.17152		

Significant (*p* ˂ 0.05), *** very highly significant, * significant, *NS* No significant

When AMOVA was performed, at three hierarchical levels, with two mountain range type, very low genetic differentiation was observed between groups (FCT = 0.066) even though that it is slightly significant (Table 4). This indicates that mountain range type has had little effect on populations’ structuration which implies that there is no local adaptation of studied populations. Also, a very low genetic differentiation was obtained between mountain versant type groups of populations (FCT =  −0.244) and among altitude groups (FCT =  −0.222).

### Genetic relationship

The pairwise F_ST_ values and geographic distances between the 12 populations are presented in Table 5. According to the Mantel test (*r* = 0.507; *P* < 0.0001), the geographical distances between populations are correlated with their corresponding genetic distances. These results suggest that the populations’ structure follows a model of isolation by geographical distance. By contrast, no significant association was obtained between the genetic distance and difference of altitude between populations (*r* =  −0.16, *P* = 0.19). Thus, the populations “AAS” and “FMJ” with a low difference of altitude (2 m) have a high value of genetic distance (0.376), and the populations “BZO” and “WAW” with a high difference of altitude (743 m) have obtained the low value of genetic distance (0.195). Between 66 pairwise F_ST_ values, 54 values are significant, meaning that the populations are widely different from each other. The significant values varied from 0.084 (FMJ/BZO; 12) to 0.466 (OUZ/KSB; 87), which mean that populations FMJ/BZO are the most genetically similar and OUZ/KSB are the most divergent. The result indicated that the Bin El Ouidane (BIN) and Bzou (BZO) populations have the lowest value of specific FST index (respectively 0.209 and 0.212), and Elksiba (KSB) population has the highest value of this index (FST = 0.289), implying that this latter population is the most divergent from the others studied. Regarding the intrapopulation variability assessed by gene diversity over loci (data not shown) which oscillated from 0.148 for Elksiba (KSB) to 0.261 for Bin El Ouidane (BIN) and 0.257 for Bzou (BZO), reflecting consequently for any in situ and/or ex situ conservation strategy should aim to include those later populations.

**Table 5:** Pairwise F_ST_ values (below diagonal) and corresponding geographic distances in km (above diagonal) and corresponding difference of altitude (in m, above diagonal in bold) for twelve populations of *E. resinifera* analyzed by ISSRs. Abbreviations as in Table 1

	KSB	TAG	AAS	OAY	AFO	MOD	BIN	BZO	FJM	IMI	WAW	OUZ
KSB	0	22 **(294)**	39 **(274)**	84 **(481)**	63 **(252)**	41**(178)**	68 **(109)**	110 **(551)**	113**(272)**	130 **(82)**	99 **(192)**	87**(104)**
TAG	0.136*	0	17 **(20)**	64 **(187)**	41 **(42)**	19 **(472)**	47 **(185)**	89 **(257)**	93 **(22)**	108**(376)**	76 **(486)**	65 **(190)**
AAS	0.292**	0.100^NS^	0	48 **(207)**	24 **(22)**	6 **(452)**	31 **(165)**	74 **(277)**	76 **(2)**	91 **(356)**	60 **(466)**	48 **(170)**
OAY	0.388**	0.187*	0.212**	0	27 **(229)**	49 **(659)**	36 **(372)**	26 **(70)**	31 **(209)**	55 **(563)**	33 **(673)**	22 **(377)**
AFO	0.375**	0.142**	0.181***	−0.014^NS^	0	24 **(430)**	15 **(143)**	51 **(299)**	52 **(20)**	67 **(334)**	37 **(444)**	25 **(148)**
MOD	0.415***	0.237**	0.287**	0.103^NS^	0.157^NS^	0	28 **(287)**	74 **(729)**	76 **(450)**	89 **(96)**	58 **(12)**	47 **(282)**
BIN	0.283***	0.146^NS^	0.194**	0.145*	0.105^NS^	0.171*	0	57 **(442)**	55 **(163)**	63 **(191)**	32 **(301)**	23 **(5)**
BZO	0.230***	0.162**	0.259***	0.253**	0.147^NS^	0.260**	0.025^NS^	0	12** (279)**	42 **(633)**	38 **(743)**	36 **(447)**
FJM	0.318***	0.255**	0.376***	0.403***	0.368**	0.414**	0.170***	0.084*	0	30 **(354)**	30 **(464)**	32 **(168)**
IMI	0.399***	0.301**	0.261***	0.269**	0.260**	0.335**	0.177^NS^	0.234***	0.342***	0	31 **(110)**	43 **(186)**
WAW	0.432***	0.234***	0.311***	0.173**	0.107*	0.301**	0.164*	0.195*	0.375**	0.147*	0	14 **(296)**
OUZ	0.466***	0.251**	0.290**	0.146*	0.064^NS^	0.233^NS^	0.186*	0.245***	0.420***	0.187***	−0.053^NS^	0
Genetic diversity ± SD	0.148 ± 0.093	0.245 ± 0.152	0.203 ± 0.127	0.190 ± 0.119	0.194 ± 0.121	0.209 ± 0.131	0.261 ± 0.162	0.257 ± 0.159	0.178 ± 0.111	0.247 ± 0.153	0.215 ± 0.134	0.192 ± 0.120

Significant (*p* ˂ 0.05): *: Significant, ** highly significant, *** very highly significant, *NS* No significant

**Fig. 2:**
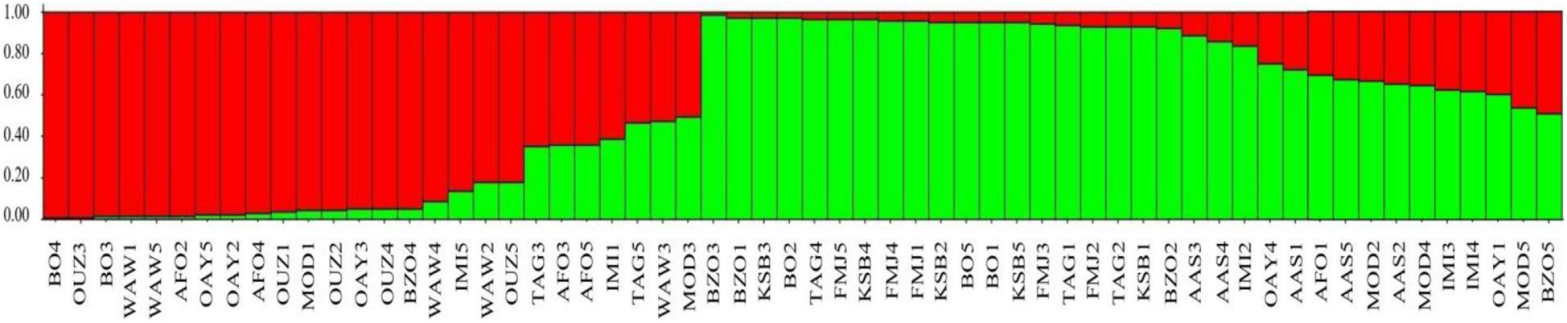
Bar plots represent STRUCTURE inferences of individual assignments (*N* = 60, *K* = 2, *ΔK* = 13.71, *H’* = 0.947). Each individual is represented by a vertical bar showing membership coefficients to each genetic cluster

The structure analysis based on the ΔK method showed that the best number of genetic clusters (K) was 2, suggesting that all individuals fell into two clusters (Fig. 2). Moreover, based on the permuted average Q-matrix generated by Clumpak, the highest H′ was observed for *K* = 2 (H′ = 0.947), indicating the stability of the result for this model. Considering the genotypes as pure when the membership coefficient was greater than 0.80 and as a hybrid or admixture when the membership coefficient was lower than 0.80, 41 individuals among the 60 analyzed (68.33%) were assigned to one of the model’s defined groups. The first group (red) was formed by the individuals from High Atlas Mountain populations, namely Bzou (BZO4), Ouled Ayyad (OAY2, OAY3, and OAY5), Bin El Ouidane (BO3 and BO4), all individuals from populations of Ouzoud (OUZ1, 2, 3, 4, and 5), Imi n’Ifri (IMI5), and four from Wawla (WAW1, WAW2, WAW4, and WAW5), with a membership coefficient oscillated between 0.815 to 0.990, while the rest of this group were originating from Middle Atlas Mountain populations: Afourer (AFO2 and AFO4), Modj (MOD1), with a membership coefficient ranging from 0.953 to 0.980. Nevertheless, the other seven individuals, namely (TAG3, TAG5) from Tagzirt population, (AFO3, AFO5) coming from Afourer population, MOD3 from Modj population, IMI1 belonging to Imi n’Ifri population, and WAW3 from Wawla population could be considered as admixed (coefficients ranged from 0.507 to 0.644). The second group (green) contained individuals coming from High Atlas Mountain populations: (BO1, BO2, and BO5) of North versant, with a membership coefficient ranging from 0.954 to 0.975, all individuals of Foum Jemaa population (FMJ1, 2, 3, 4, and 5), and one individual (IMI2) from Imi n’Ifri population belonging to Southwest versant (coefficients between 0.840 and 0.959) and (BZO1, BZO2, and BZO3) collected from South versant having a membership coefficient between 0.922 and 0.986, while the rest of group include bushes coming from Middle Atlas Mountain populations: (AAS3 and AAS4) of North versant with an assignment coefficient of 0.888 and 0.86 respectively, (TAG1, TAG2 and TAG4) of Southwest versant with a membership coefficient from 0.932 to 0.969, and all bushes of Elksiba population (KSB1, 2, 3, 4, and 5) from South versant mountain, having a coefficient of assignment oscillating between 0.929 and 0.976. Finally, 12 remaining bushes which could be considered as admixture belonged to the OAY, AAS, AFO, MOD, IMI, and BZO populations (coefficients between 0.508 and 0.775).

**Fig. 3:**
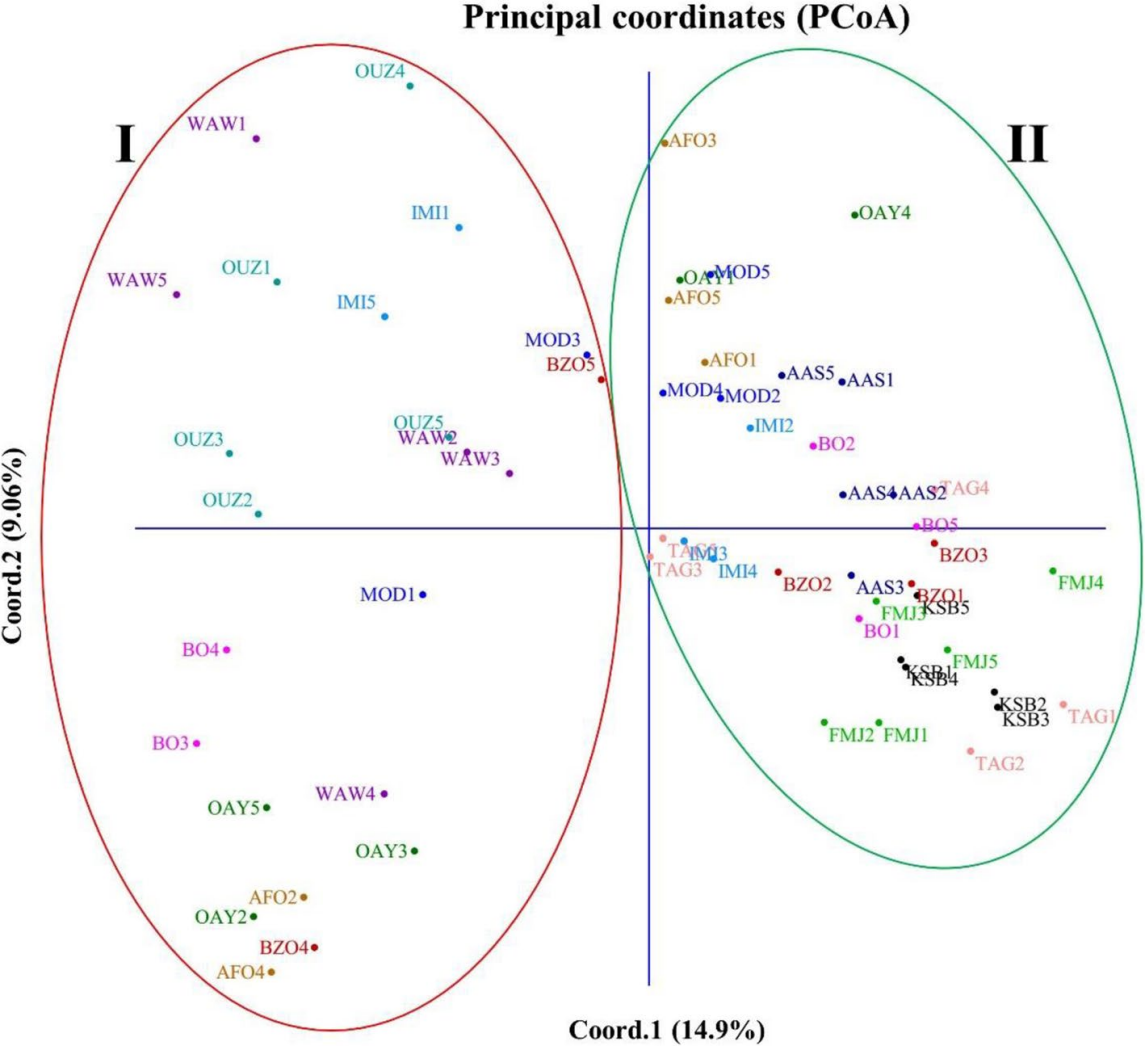
PCoA plot of the *E. resinifera* individuals showing relationships based on ISSR data

The genetic structure of Moroccan *E. resinifera* populations was further reconstructed by using the PCoA. Indeed, about 24% of total variance was explained by the first two components and the plot of PCoA divided studied populations in two groups (I and II, Fig. 3), which corroborates the populations’ structure obtained by Bayesian analysis (Fig. 2). Consequently, the genetic structure of all studied populations bushes in two main groups was operated independently of altitude and mountain versant type.

## Discussion

DNA markers have become a useful tool for evaluating the genetic diversity of many different plant species. In this work, ISSR markers were used to assess the genetic diversity of *E. resinifera* populations in Morocco. The 14 tested ISSRs primers revealed a high percentage of polymorphism with an average of 74.81%. This high percentage implies that there is an important genetic diversity in this endemic species. This result is higher than that reported with ISSR markers for other *Euphorbia* species (*E. khabrica*, *E. buhsei*, *E. osyridea*, and *E. austro-iranica*) in Iran by Pahlevani et al. (13.02%, 20.71%, 24.85%, and 14.20%, respectively) [50], in Saudi Arabia by Moustafa et al. (*Euphorbia prostrata Aiton*, *Euphorbia peplus L.*, and *Euphorbia terracina L.*) (20.28%, 14.08%, and 11.44%, respectively) [51], and for 15 *Euphorbia* species by El-Hawary et al. (total polymorphism = 57.7%) [52]. However, our finding was lower than that observed by Reginaldo et al. for Brazilian *Euphorbiaceae* (*Croton urucurana Baill*) (89%) based on ISSR markers [53] and by Dorset et al. for American *E. telephioides* (80.7%) revealed by allozyme markers [54]. In addition, the high values of PIC (0.33) and Rp (2.8) parameters show that the ISSR markers are very informative and efficient for analyzing the diversity and genetic structure of *E. resinifera*. These values are comparable with these obtained by Reginaldo et al. for Brazilian *Euphorbiaceae* (*Croton urucurana Baill*) using ISSRs markers (PIC = 0.29 and Rp = 3.4) [53]. Moreover, the high multi-locus value of Ht (= 0.21) suggests the presence of a high level of polymorphism of this endemic species. Indeed, this high polymorphism is confirmed by the Shannon index value (*I* = 0.35). This value is higher than that obtained by Reginaldo et al. (*I* = 0.26) for Brazilian *Euphorbiaceae* using ISSRs markers [53]. Besides, the gene diversity within the species (Hs) was 0.15. This high genetic diversity obtained in Moroccan *E. resinifera* populations is in agreement with the general trend for allogamous and long-lived woody perennial species (Ht = 0.28) and for angiosperm species (Ht = 0.28) [55]. The wild populations of *E. resinifera* were largely differentiated (Gst = 0.32, FST = 0.244) which could be due to restricted gene flow (N_e_m = 0.77) between populations. This finding is in concordance with that observed for other herbaceous outcrossing perennial plant species (Gst > 0.20) [56]. The Gst value (0.32) detected in this investigation is higher than that recorded by Dorset et al. for the endemic North American species *Euphorbia telephioides* (0.10) using allozyme markers [54]. Similar results were revealed by Ki-Ryong for *Euphorbia fauriei* in Korea (Fst = 0.237; N_e_m = 0.80) and *Euphorbia jolkinii* in Taiwan (Fst = 0.245; N_e_m = 0.77) [41]. The indirect estimate of gene flow on the basis of FST was low (Nem = 0.77) and might be due to the presence of geographical barriers between populations such mountains. Indeed, many factors such as discontinuous distribution of populations, limited pollinator movements, and low rate of seed migration could be an efficient obstacle to the gene flow and the origin of the high value of differentiation among *E. resinifera* populations.

The analysis of molecular variance (AMOVA) showed that 75.56% of the diversity accounted for within population leaving 24.44% for among populations. The existence of high genetic variability within population should help the population to cope with local environmental changes. This result implies that sampling from a small number of populations, particularly those with high intrapopulation variability, is sufficient for species in situ and/or ex situ conservation. Bin El Ouidane (BIN) and Bzou (BZO) populations with high intrapopulation variability are more convenient for this purpose.

Moreover, hierarchical AMOVA revealed a very low genetic differentiation between the two groups of mountain range (FCT = 0.066), even though that it is slightly significant, which suggests that mountain range type have had little influence on structuration’s populations. These findings give choice to sampling from populations of Middle or High Atlas Mountains for conservation and breeding species. Likewise, when assembling the populations according to their mountain versant type and altitude, a very low genetic differentiation was obtained between respective groups (respectively, FCT =  −0.024 and FCT =  −0.022), indicating that mountain versant type and altitude did not have an effect on population structure of the Moroccan *E. resinifera* populations. Strangely enough, genetic distances were not correlated to the difference of altitude between the populations (*r* =  −0.16, *P* = 0.19). A similar result was reported for Moroccan walnut using SSR marker [57]. Also, other studies described no genetic differentiation among populations at low and high altitudes [58, 59], probably due to the overlap of flowering phenology in populations at different altitudes, species’ extensive pollen flow, and long distance seed dispersal among different altitudes by animals especially birds. This result is confirmed by the biased model and PCoA which showed that the twelve populations are gathered in two groups undependably to mountain range, mountain versant type, and altitude. In contrast, geographic distances have explained the genetic differentiation between populations according to the Mantel test (*r* = 0.507; *P* < 0.0001). This result suggests that the population structure follows a model of isolation by geographic distance. Consequently, our finding suggests that more closely situated populations tend to be more genetically similar to one another.

## Conclusion

The present study is the first work aiming to evaluate the genetic diversity and structure of *E. resinifera* populations in Morocco using ISSR markers. The results of this study confirmed that ISSR markers could be powerful tools for detecting genetic diversity among and within *E. resinifera* populations. The level of genetic diversity was high, and the genetic variation mainly existed within populations. The results led to structure of populations in two gene pools independently of mountain range type, mountain versant type, and altitude. Based on these results, it could be sufficient to sample from a few populations, particularly those most genetically diversified, for any in situ and/or ex situ conservation strategy.

## Data Availability

All data generated or analyzed during this study are included in this article.

## Abbreviations

ISSR: Inter simple sequence repeat
PIC: Polymorphic information content
Rp: Resolving power
I: Shannon’s information index
Ht: Total genetic diversity
Hs: Genetic diversity within populations
PCR: Polymerase chain reaction
DNA: Deoxyribonucleic acid
TBE: Tris-Borate-EDTA
UV: Ultraviolet
PPB: Percentage of polymorphic band
Na: Numbers of alleles
Ne: Effective number of alleles
Gst: Coefficient of gene differentiation
AMOVA: Analysis of molecular variance
N_e_m: Gene flow
UPGMA: Unweighted pair-group method with arithmetic mean
MCMC: Markov chain Monte Carlo
FST: Pairwise genetic differentiations
UBC: University of British Columbia
